# Examining the relationship between inflammatory biomarkers during COVID‐19 hospitalization and subsequent long‐COVID symptoms: A longitudinal and retrospective study

**DOI:** 10.1002/iid3.1052

**Published:** 2023-10-30

**Authors:** Dominic L. Sykes, Christina M. Van der Feltz‐Cornelis, Luke Holdsworth, Simon P. Hart, Joseph O'Halloran, Steve Holding, Michael G. Crooks

**Affiliations:** ^1^ Hull York Medical School Hull UK; ^2^ Department of Health Sciences University of York York UK; ^3^ Hull York Medical School York United Kingdom; ^4^ Institute of Health Sciences University College London London United Kingdom; ^5^ Buckinghamshire Healthcare NHS Trust Aylesbury United Kingdom; ^6^ NHS Frimley Health Foundation Trust Frimley United Kingdom

**Keywords:** biomarkers, COVID‐19, interleukin‐6, long‐COVID

## Abstract

**Introduction:**

Long‐COVID is a heterogeneous condition with a litany of physical and neuropsychiatric presentations and its pathophysiology remains unclear. Little is known about the association between inflammatory biomarkers, such as interleukin‐6 (IL‐6) and C‐reactive protein (CRP) in the acute phase, and persistent symptoms after hospitalization in COVID‐19 patients.

**Methods:**

IL‐6, CRP, troponin‐T, and ferritin were analyzed at admission for all patients with COVID‐19 between September 1, 2020 to January 10, 2021. Survivors were followed up 3‐months following hospital discharge and were asked to report persistent symptoms they experienced. Admission data were retrospectively collected. Independent *t*‐tests and Mann–Whitney *U* tests were performed.

**Results:**

In a sample of 144 patients (62.5% male, mean Age 62 years [SD = 13.6]) followed up 3 months after hospital discharge, the commonest symptoms reported were fatigue (54.2%), breathlessness (52.8%), and sleep disturbance (37.5%). In this sample, admission levels of IL‐6, CRP and ferritin were elevated. However, those reporting myalgia, low mood, and anxiety at follow‐up had lower admission levels of IL‐6 (34.9 vs. 52.0 pg/mL, *p* = .043), CRP (83 vs. 105 mg/L, *p* = .048), and ferritin (357 vs. 568 ug/L, *p* = .01) respectively, compared with those who did not report these symptoms. Multivariate regression analysis showed that these associations were confounded by gender, as female patients had significantly lower levels of IL‐6 and ferritin on admission (29.5 vs. 56.1, *p* = .03 and 421.5 vs. 589, *p* = .001, respectively) and were more likely to report myalgia, low mood and anxiety, when compared to males.

**Conclusions:**

Our data demonstrate that female patients present more often with lower levels of inflammatory biomarkers on admission which are subsequently associated with long‐term post‐COVID symptoms, such as myalgia and anxiety, in those discharged from hospital with severe COVID‐19. Further research is needed into the role of serum biomarkers in post‐COVID prognostication.

## Graphical abstract

Among severe acute COVID‐19 survivors, the inflammatory profile at the time of hospital admission may be associated with future long‐COVID symptomatology. Further research is needed into the role of serum biomarkers in post‐COVID prognostication.

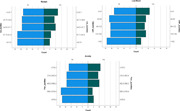

## 1 INTRODUCTION

The importance of measuring inflammatory biomarkers in patients hospitalized with COVID‐19 is well established.^1^ Early reports described worse outcomes in patients with higher serum levels of inflammatory serum biomarkers, evidenced by increased risk of critical care admission and inpatient mortality.^2^ Many serum biomarkers have been studied in relation to COVID‐19, including interleukin‐6 (IL‐6), a pro‐inflammatory cytokine produced by macrophages that has been shown to play a role in the pathophysiology of COVID‐19, particularly in severe cases.^3^

The lasting impact of COVID‐19 has become widely recognized and is known under the collective term long‐COVID. The associated morbidity places a significant burden on those affected and health systems worldwide.^4^ Long‐COVID is a markedly heterogeneous condition with a litany of physical and neuropsychiatric presentations, which include breathlessness, fatigue, “brain fog,” anxiety, and myalgia.^5^, ^6^ The largest prospective study examining persistent post‐COVID‐19 symptoms, PHOSP‐COVID, has investigated inflammatory profiles amongst those with long‐COVID.^7^ This study examined a broad range of proteins shown to be elevated in those who report severe persistent symptoms, including IL‐6, CD70, and hepatocyte growth factor (HGF) and has found some of these proteins to be persistently elevated at five months post‐discharge. There is a relative dearth of studies examining the association of admission inflammatory profiles with long‐term symptoms. For symptoms such as anxiety, low mood, and “brain fog,” there is an ongoing debate about whether they are caused by ongoing inflammatory or vascular processes or by distress due to prolonged COVID‐19 symptoms.^8^

In this study, we examined inflammatory biomarkers (IL‐6, Ferritin, CRP, and Troponin) measured at the point of hospitalization with severe acute COVID‐19 and the development of long‐COVID in survivors to explore the possibility of an association.

## 2 METHODS

### 2.1 Study design

Data were collected for all patients admitted with acute COVID‐19 between September 1, 2020 and January 10, 2021 to two large University Teaching Hospitals within a single NHS Hospital Trust in Yorkshire, England. An automated alert system was established within the hospital laboratories, prompting the following panel of tests to be undertaken on patients' blood samples taken at the time of hospital admission, when a nasopharyngeal swab confirmed SARS‐CoV‐2 infection using RT‐PCR: C‐reactive protein (CRP), ferritin, IL‐6 and troponin‐T. This was part of the standard investigation pathway for patients admitted with COVID‐19 and results were made available to treating clinicians. No additional blood samples were taken from patients as part of this analysis.

Biomarker analysis was performed on pre‐existing routine clinical blood samples of patients admitted with COVID‐19 by biochemistry laboratory staff. Serum ferritin and CRP were measured by Beckman Coulter latex enhanced turbidimetry using AU5800 analyzers (Beckman Coulter Inc). Serum troponin‐T (high‐sensitivity assay) and IL‐6 were measured by Roche Elecsys biotin‐streptavidin based electrochemiluminescence assays using a e411 analyzer (Roche Diagnostics). All assays were performed in accordance with the manufacturers' recommended methods.

All survivors that had COVID‐19 pneumonia confirmed on chest imaging during their admission were offered follow‐up approximately 3 months following hospital discharge as part of their routine clinical care, patients were asked to report any new symptoms that they had been experiencing since their admission. Persistent symptoms were recorded using a standardized pro‐forma and validated symptoms (Medical Research Council [MRC] Dyspnoea Scale) and quality of life questionnaires (5‐level EuroQol‐5 Dimension [EQ‐5D‐5L]) were undertaken. Those receiving follow‐up through this service were included in analysis. Data for patients with severe frailty or those resident in care homes were unavailable as their follow‐up was undertaken by third‐party specialist frailty services within the community and are therefore not included in our analysis.

Data were collected retrospectively from patients' electronic health records. The following data were collected relating to participants' hospitalization: age, sex, co‐morbidities, date of onset of symptoms, admission respiratory rate, oxygen requirement on admission, Glasgow Coma Score, length of stay, ICU admission, death. Serum biomarkers recorded were CRP, alanine aminotransferase (ALT), urea, IL‐6, Troponin‐T, lymphocytes, neutrophils, platelets, D‐Dimer. For those attending follow‐up, the following additional data were collected: MRC Dyspnoea Scale, EQ‐5D‐5L, and the presence or absence of a range of symptoms described in the long‐COVID literature.

Comparison of means were performed using independent *t*‐testing, comparison of medians were performed using Mann–Whitney *U* testing. A multivariate logistic regression analysis was also performed, controlling for gender and age. Statistical analyses were performed using IBM SPSS Statistics 26.0 (Armonk, NY: IBM Corp) and GraphPad Prism version 9.0.0.

## 3 RESULTS

### 3.1 Symptom burden at follow‐up

**Figure 1:**
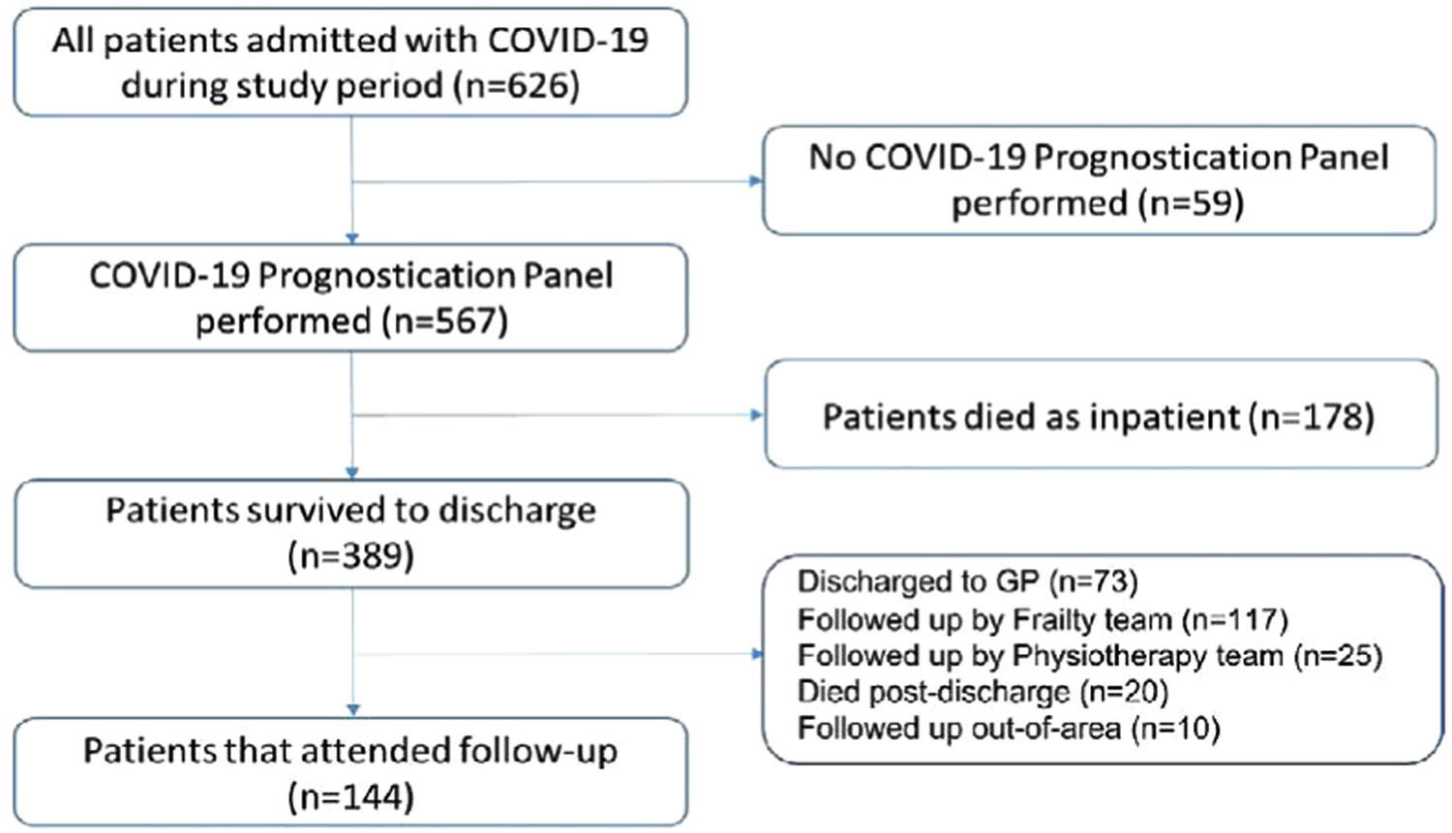
Flowchart of patients admitted to hospital and their follow‐up pathway.

In total, 389 patients survived to discharge and 144 patients with complete biomarker and 3‐month follow‐up symptom data were included in the final analysis. Reasons for exclusion of 245 patients from the final analysis were: 20 patients died after discharge but before follow‐up, 73 were discharged to GP for follow‐up, and 152 patients were followed‐up by alternative clinical services and therefore follow‐up data were unavailable (117 by local frailty services, 25 by a complex rehabilitation team, and 10 patients outside of the local area). Figure 1 shows the flowchart.

Of the 144 patients followed up for symptom assessment, 62.5% (*n* = 90) were males and the mean age of the cohort was 62 years (SD = 13.6). All baseline demographics and admission data can be found in Table 1.

**Table 1:** Baseline characteristics, admission data, and admission biochemical markers in survivors with follow‐up assessment.

	All patients (*n* = 144)	Females (*n* = 54)	Males (*n* = 90)	*p*‐Value
Age (SD)	62.0 (13.6)	65.3 (11.9)	60.9 (14.3)	.088
BMI (SD)	30.1 (6.8)	30.2 (8.5)	30.1 (5.8)	.958
*Comorbidities (%)*				
Type 1 diabetes	2 (1.3)	2 (3.7)	0 (0)	.066
Type 2 diabetes	36 (25.0)	8 (14.8)	28 (31.1)	.029
Ischemic heart disease	4 (2.8)	0 (0)	4 (4.4)	.116
COPD	20 (13.9)	10 (18.5)	10 (11.1)	.213
Asthma	28 (19.4)	9 (16.7)	19 (21.1)	.583
Hypertension	61 (42.4)	25 (46.3)	36 (40.0)	.459
CKD	4 (2.8)	2 (3.7)	2 (2.2)	.600
History of VTE	5 (3.5)	0 (0)	5 (5.5)	.078
Smoking history	83 (57.6)	29 (53.7)	54 (60.0)	.459
Alcohol use	82 (56.9)	20 (37.0)	62 (68.9)	**<.001**
*Maximum oxygen/respiratory support requirement (%)*				
Air	30 (20.8)	16 (29.6)	14 (15.5)	.07
Nasal cannula/face mask	87 (60.4)	29 (53.7)	58 (64.4)	.63
CPAP/BIPAP	12 (8.3)	5 (9.3)	7 (7.8)	.76
High flow nasal cannula	14 (9.7)	4 (7.4)	10 (11.1)	.47
Intubation and ventilation	1 (0.7)	0 (0)	1 (0.1)	‐
*Median admission prognostication panel markers (range)*				
Interleukin‐6 (pg/mL)	45.9 (1.5–347.4)	29.5 (1.6–264.8)	56.1 (1.5–347.4)	**.003**
C‐reactive protein (mg/L)	96 (0.7–380)	88 (0.7–304)	115 (2.3–380)	**.066**
Troponin T (ng/L)	12.5 (5–318)	11.5 (7–49)	13 (5–318)	.381
Ferritin (μg/L)	533.5 (27–3311)	421.5 (27–1459)	589 (29–3311)	**.001**

*Note*: Comparisons of means were performed with *t*‐tests, comparisons of medians were performed with Mann–Whitney *U* tests, comparisons of proportions were performed with chi‐squared tests. Abbreviations: BiPAP, bilevel positive airway pressure; BMI, body mass index; CKD, chronic kidney disease; COPD, chronic obstructive pulmonary disease; CPAP, continuous positive airway pressure; VTE, venous thromboembolism. *p*‐values in bold are significant.

The commonest symptoms reported by patients at follow‐up were fatigue (54.2%), breathlessness (52.8%), sleep disturbance (37.5%), cough (35.4%), and memory impairment (30.6%). In our cohort, anxiety was significantly more common in females than males (37% vs. 21.1%, *p* = .021). Myalgia was also more commonly found in females than males (40.7% vs. 22.2%, *p* = .018). All symptom follow‐up data can be found in Table 2.

**Table 2:** Frequency of symptoms reported for all patients and split by gender at follow‐up, and significance of gender differences.

	All patients (*n* = 144)	Female (*n* = 54)	Male (*n* = 90)	*p*‐Value
*Symptoms at follow‐up (N, %)*				
Breathlessness	76 (52.8)	31 (62.0)	45 (50.0)	.389
Myalgia	42 (29.2)	22 (40.7)	20 (22.2)	**.018**
Anxiety	40 (27.8)	21 (37.0)	19 (21.1)	**.021**
Extreme fatigue	78 (54.2)	33 (61.1)	45 (50.0)	.195
Low mood	38 (26.4)	16 (29.6)	22 (24.4)	.494
Memory impairment	44 (30.6)	21 (37.0)	23 (25.6)	.093
Sleep disturbance	54 (37.5)	21 (37.0)	33 (36.7)	.790
Cough	51 (35.4)	22 (40.7)	20 (22.2)	.301
Attention deficit	19 (13.2)	7 (13.0)	12 (13.3)	.949
Pleuritic chest pain	27 (18.8)	5 (9.3)	5 (5.6)	.408
Sore throat	10 (6.9)	10 (18.5)	7 (7.8)	.397
Fever	6 (4.2)	1 (1.9)	8 (8.9)	.282
Anosmia	24 (16.7)	12 (22.2)	12 (13.3)	.166
Taste deficiency	28 (19.4)	17 (18.5)	11 (12.2)	**.005**
Rash	14 (9.7)	7 (13.0)	7 (7.8)	.309
*Median MRC breathlessness scale score (range)*				
Before	1 (1–5)	1 (1–5)	1 (1–5)	.113
After	2 (1–5)	2 (1–5)	2 (1–5)	.111

*Note*: Comparisons of medians were performed with Mann–Whitney *U* tests, comparisons of proportions were performed with chi‐squared tests. *p*‐values in bold are significant.

### 3.2 Associations between admission biomarkers and persistent symptoms at follow‐up

Table 3 shows admission inflammatory markers for patients that did and did not report individual symptoms at follow‐up.

**Table 3:** Median levels of inflammatory markers in patients reporting symptoms at follow‐up.

	Median IL‐6 [pg/mL] (range)		Median CRP [mg/L] (range)	Median ferritin [μg/L] (range)	Median troponin T [ng/L] (range)
**Symptoms at follow‐up (*n*)**					
*Breathlessness*					
Yes (*n* = 76)	43.2 (2.7–347.4)		86.5 (2.6–361)	516 (28–3311)	11 (5–318)
No (*n* = 68)	48.5 (1.5–338.5)		104 (0.7–380)	545 (27–2765)	13.5 (5–118)
*p*‐Value	.885		.974	.916	.093
*Myalgia*					
Yes (*n* = 42)	**34.9** (1.6–338.5)		101 (0.7–304)	457.5 (28–3183)	12.5 (5–318)
No (*n* = 102)	**49.5** (1.5–347.4)		90.5 (2.3–380)	546.5 (27–3311)	12.5 (5–118)
*p*‐Value	**.043**		.772	.229	.772
*Anxiety*					
Yes (*n* = 40)	42.7 (2.7–191.2)		92.5 (0.7–244)	**345.5** (27–3183)	12 (5–318)
No (*n* = 104)	48.5 (1.5–347.4)		103.5 (2.3–380)	**572** (28–3311)	13 (6–118)
*p*‐Value	.495		.248	**.016**	.417
*Extreme fatigue*					
Yes (*n* = 78)	40.7 (1.6–347.5)		92 (0.7–354)	469 (27–3311)	12 (5–318)
No (*n* = 66)	52.9 (1.5–264.8)		104 (9–380)	569 (63–2765)	13 (5–49)
*p*‐Value	.221		.777	.115	.794
*Low mood*					
Yes (*n* = 38)	34.9 (2.2–191.2)		**79.5** (0.7–304)	**345.5** (27–3183)	12 (5–318)
No (*n* = 106)	52 (1.5–347.4)		**105** (2.3–380)	**568** (53–3311)	13 (6–169)
*p*‐Value	.068		**.048**	**.010**	.426
*Memory impairment*					
Yes (*n* = 44)	44.4 (13.4–338.5)		83 (2.6–304)	457 (29–3183)	12 (5–318)
No (*n* = 100)	46.6 (1.5–347.4)		105 (0.7–380)	546 (27–3311)	12.5 (5–118)
*p*‐Value	.687		.647	.510	.267
*Sleep disturbance*					
Yes (*n* = 54)	48.5 (1.6–347.4)		110 (2.3–354)	539 (29–3183)	13 (7–318)
No (*n* = 90)	41.2 (1.5–338.5)		89 (0.7–380)	516 (27–3311)	12 (5–118)
*p*‐Value	.266		.525	.583	.401
*Cough*					
Yes (*n* = 51)	45.3 (1.5–191.2)		103 (11–307)	447 (27–3311)	13 (7–318)
No (*n* = 93)	76.7 (1.6–347.4)		89 (0.7–380)	550 (28–2765)	12 (5–118)
*p*‐Value	.474		.737	.679	.752
*Attention deficit*					
Yes (*n* = 19)	43.4 (16.4–191.2)		93 (2.6–244)	550 (29–3183)	11 (5–318)
No (*n* = 125)	46.4 (1.5–347.4)		99 (0.7–380)	528 (27–3311)	13 (5–118)
*p*‐Value	.752		.639	.302	.370
*Pleuritic chest pain*					
Yes (*n* = 27)	51.5 (14.8–191.2)		67 (20–244)	313 (32–1269)	11.5 (5–169)
No (*n* = 117)	43.2 (1.5–347.4)		105 (0.7–380)	568 (27–3311)	13 (5–318)
*p*‐Value	.512		.666	.080	.216
*Sore throat*					
Yes (*n* = 10)	41.9 (1.6–191.2)		104.5 (4.2–244)	330.5 (32–1030)	11.5 (7–74)
No (*n* = 134)	46.1 (1.5–347.4)		92 (0.7–380)	539.5 (27–3311)	13 (5–318)
*p*‐Value	.881		.825	.175	.975
*Fever*					
Yes (*n* = 6)	28.4 (23.5–191.2)		77 (23–380)	325 (53–1673)	13 (8–16)
No (*n* = 138)	46 (1.5–347.4)		101 (0.7–361)	539.5 (27–3311)	12.5 (5–318)
*p*‐Value	.940		.640	.520	.924
*Anosmia*					
Yes (*n* = 24)	43.2 (16.4–191.2)		96 (4.2–285)	366 (28–3183)	10.5 (7–169)
No (*n* = 120)	45.9 (1.5–347.4)		97.5 (0.7–380)	550 (27–3311)	13 (5–318)
*p*‐Value	.768		.142	.221	.910
*Taste deficiency*					
Yes (*n* = 28)	44.9 (12–191.2)		96 (2.6–361)	473 (28–1599)	11.5 (7–169)
No (*n* = 116)	46.1 (1.5–347.4)		97.5 (0.7–380)	546.5 (27–3311)	13 (5–318)
*p*‐Value	.762		.649	.553	.836
*Rash*					
Yes (*n* = 14)	33.5 (9.6–181.6)		96 (2.6–197)	255.5 (29–3183)	10.5 (7–169)
No (*n* = 130)	46.6 (1.5–347.4)		97.5 (0.7–380)	546.5 (27–3311)	13 (5–318)
*p*‐Value	.356		.358	.30	.696

*Note*: Comparisons of medians were performed with Mann–Whitney *U* tests. Significant findings are in bold. *p*‐values in bold are significant.

Multivariate analyses for myalgia and anxiety are shown in Supporting Information.

#### 3.2.1 IL‐6

Those reporting myalgia at follow‐up had a significantly lower median IL‐6 level compared with those not reporting this symptom (34.9 vs. 49.5 pg/mL), *p* = .043). Figure 1 displays a significant association with higher prevalence of myalgia in those with lower baseline serum IL‐6 level. However, on performing multivariate regression it was found that this association was confounded by gender, with females being significantly more likely to report myalgia, when compared to males (*p* = 0.032).

#### 3.2.2 CRP

**Figure 2:**
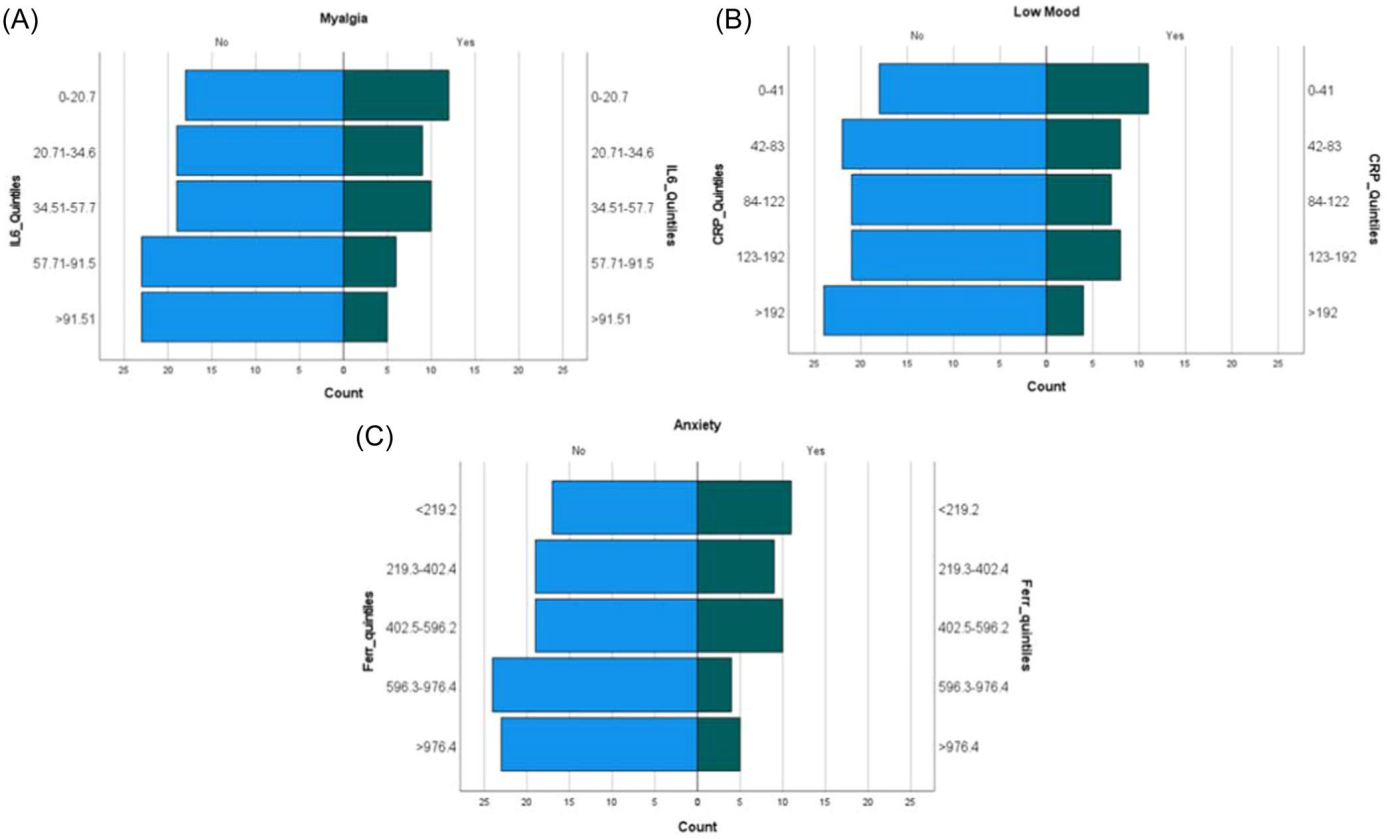
Population pyramids demonstrating the prevalence of symptoms at follow‐up split by quintiles of inflammatory biomarkers. (A) Displaying the significant association with lower prevalence of myalgia in those with higher baseline interleukin‐6 (IL‐6). (B) Showing the lower prevalence of low mood associated with higher levels of baseline C‐reactive protein (CRP). (C) Displaying the lower prevalence of anxiety in those with higher levels of ferritin at baseline.

Those who reported low mood at follow‐up had a significantly lower median CRP level on admission than those who did not (79.5 vs. 105 mg/L, *p* = .048). Figure 2 demonstrates the prevalence of symptoms at follow‐up split by quintiles of inflammatory biomarkers. This shows a higher prevalence of low mood associated with lower levels of serum CRP at admission. On multivariate regression analysis, the association between lower levels of CRP and presence of low mood did not reach significance (*p* = .083).

#### 3.2.3 Ferritin

Those who reported anxiety at follow‐up had a significantly lower median level of ferritin on admission than those who did not (345.5 vs. 572 μg/L, *p* = .016). Figure 1 conveys a lower prevalence of anxiety in those with higher levels of ferritin at baseline. Those who reported low mood at follow‐up also had a significantly lower median level of ferritin on admission than those who did not (345.5 vs. 568 μg/L, *p* = .01). However, on performing multivariate regression it was found that this association was confounded by gender, as females were more likely to report anxiety (*p* = 0.050).

#### 3.2.4 Troponin‐T

There were no differences in levels of troponin‐T in patients who reported symptoms of any kind in this study.

#### 3.2.5 Nature of admission

There were no associations between symptom burden at follow‐up and length of stay as an inpatient. There were also no associations between increased symptom burden and more severe illness, as there were no differences in those treated on standard medical wards, high‐dependency units, or intensive care units.

## 4 DISCUSSION

Our findings demonstrate that those reporting myalgia, low mood, and anxiety at follow‐up had lower admission IL‐6, CRP, and ferritin respectively, than those without these symptoms in the group that survived. However, these associations were not found when adjusted for age and gender. Our data show that females were significantly more likely to reports such symptoms and had significantly lower levels of IL‐6 and ferritin on admission (29.5 vs. 56.1, *p* = .03 and 421.5 vs. 589, *p* = .001, respectively). This may suggest that those with lower grade inflammation at admission could be at higher risk of persistent neuropsychiatric symptoms. The authors believe that females may be at higher risk at persistent low‐grade inflammation compared to males. There have been previous findings of systemic low‐grade inflammation in psychiatric disorders in the literature,^9^ which has various implications for treatment and clinical outcomes.^10^, ^11^ Furthermore, we have also corroborated the evidence from previous work^5^ that female patients are significantly more likely to report neuropsychiatric symptoms in the post‐COVID‐19 period, as in this study anxiety and myalgia were significantly more common in females.

Persistent neuropsychiatric symptoms are commonly reported in those with long‐COVID. Meta‐analysis data show the commonest symptoms to be sleep disturbance, anxiety, objective cognitive impairments, and fatigue.^12^, ^13^ Our results are concordant with such findings and with previous studies regarding the lack of association with severity of acute illness and symptom burden at follow‐up. Indeed, there are data to suggest a higher prevalence of persistent symptoms in patients who were not hospitalized with COVID‐19.^13^, ^14^ Studies have shown, largely through online surveys, that patients who have a mild illness in the community report symptoms of long‐COVID up to 6 months after the acute illness.^15^ Long‐COVID is clinically heterogenous and the mechanism underlying the range of reported physical and neuropsychiatric symptoms remains unclear. Our study cannot exclude a lower level, persistent inflammatory process during COVID‐19 convalescence.

The association of inflammatory biomarkers during acute COVID‐19 presentation with persistent symptoms requires further study, as there has been little data examining their prognostic value in this regard. Recent study has demonstrated that lower levels inflammatory biomarkers including neutrophil counts and platelets were associated with dyspnoea or fatigue at follow‐up.^16^ Further work demonstrated no association between raised inflammatory biomarkers such as IL‐6 and CD25, measured at 10 weeks follow‐up, and post‐COVID symptoms.^17^ However, data from the PHOSP‐COVID study has shown elevated levels of IL‐6, amongst other proteins, at 5‐months in those with persistent cognitive symptoms. That study also explored the potential role of inflammatory proteins such as CD70 which have previously been associated with central nervous system (CNS) inflammation, as both IL‐6 and CD70 were raised in patients with cognitive impairment at 1‐year follow‐up.^7^ There seems to be an association of low inflammation in the acute phase with persistent neuropsychiatric symptoms, as indicated in this sample by anxiety and low mood. This aligns with literature indicating an association between low mood and anxiety and low‐grade systemic inflammation, although the mechanism is unknown. One plausible cause could be due to cell activation from inflammatory markers.^18^ Additionally, since cases of depression are a lot higher in those patients who are ill, who also have a confounding inflammation, it could be assumed that inflammation could exacerbate depression and other mental illness in Long COVID as well. Furthermore, there is an association between increased stressors and increased mental illness, for instance, uncontrollable events like deaths in the family are powerful triggers for depression; and such might very well have occurred, especially in the first phase of the pandemic.^19^ The possibility of inflammation playing a role in the pathophysiology of neuropsychiatric symptoms in Long COVID could be influenced as well by how trauma and adverse events have such a detrimental effect on somebodies health. It is commonly known that stress can induce inflammation,^20^, ^21^, ^22^ by causing imbalances to the homeostatic mechanism, ultimately causing the release of glucocorticoids via the hypothalamic‐pituitary‐adrenal axis. These stressors can be caused by physiological stimuli, such as infection or temperature changes; and by psychological pressures, like a variety of adverse events in the context of the pandemic.^23^ This paints a mixed picture, and further investigation is needed into the interplay between the inflammatory response in the acute phase of COVID‐19 and the persistent, lower‐level inflammation observed in patients with long‐COVID and anxiety or depression.

The current understanding of the relationship between biomarkers and persistent symptoms is unclear and our study was limited by the single measurement of inflammatory biomarkers at admission to hospital. It may therefore be important to examine serial levels of such biomarkers over the course of the acute illness as well as at follow‐up to elucidate their role further in this complex pathophysiology and to establish potential for a clinical application for such biomarkers for prognostication. In terms of generalizability of our findings, this study was conducted in patients admitted to hospital with COVID‐19 and therefore the findings may not be applicable for patients with COVID‐19 in the community. Furthermore, the availability of the first vaccines for COVID‐19 was limited during our study periods, only becoming available in the UK in December 2020, therefore the effect of vaccination on development of persistent symptoms cannot be accounted for in this study. This study is largely hypothesis‐generating in nature. Further research would be needed to explore the clinical relevance of profiling with biomarkers in the acute phase for Long COVID symptoms, potentially through collaboration with established, aligned, large‐scale research programmes.^24^

### 4.1 Limitations of the study

Our study represents a patient cohort from two hospitals within a single NHS trust in Yorkshire and focused on a specific subgroup of the COVID‐19 survivors who were hospitalized during an early pandemic time frame, which may not fully represent the entire population of COVID‐19 patients. This may limit the generalizability of the results to other settings and populations.

Since the admission data was collected retrospectively from the electronic records, there is a possibility of bias due to missing data, inconsistent data collection practices, and incomplete documentation that could have affected the accuracy of the results. However, the routine measurement of IL‐6, CRP, ferritin, and troponin‐T in all consecutive patients admitted with symptomatic COVID‐19 would minimize selection bias for our follow‐up symptom burden analyses. Also, the follow‐up data were assessed systematically during follow‐up visits albeit with a relatively simple assessment of persistent symptoms; we were only able to detect the presence or absence of symptoms and could not ascertain the severity of individual symptoms using validated questionnaires. Furthermore, despite the authors being able to collect data on common comorbidities of physical conditions associated with mortality in COVID‐19 such as cardiovascular and respiratory disease, there was no routine data collected for neurological or psychiatric disease. Therefore, we were not able to examine the association of previous psychiatric history and neuropsychiatric illness at follow‐up.

The study did not include a control group of non‐COVID‐19 patients, which makes it challenging to determine whether the symptoms observed were specific to COVID‐19 or common in patients after discharge from hospital for respiratory tract infections.

The study's sample size was relatively small, which might limit the statistical power of the study and make it challenging to reach definitive conclusions. The number of statistical comparisons in this study raises the possibility of associations being statistically significant “by chance.” However, the concordance of the comparisons of medians and the population pyramids combined with the consistency of the findings of lower levels of different inflammatory biomarkers in symptoms that are known to occur in clusters may reflect that these findings are true associations. We believe that our current findings should only be interpreted as hypothesis‐generating, as we reported correlations between biomarker levels and persistent symptoms but did not establish causation. This warrants further investigation in larger, prospective replication studies to explore if biomarker profiling might help predicting which patients may require follow‐up after their acute illness.

## 5 CONCLUSIONS

Our data demonstrate that lower levels of inflammatory biomarkers on admission are associated with some long‐term symptoms, such as myalgia, anxiety, and low mood, in those discharged from the hospital with severe COVID‐19. Multivariate regression shows that these associations are confounded by age and gender. We found female participants are more likely to report symptoms such as anxiety and myalgia and have significantly lower levels of inflammatory biomarkers at admission. These results provide new insights into the relationship between inflammatory profiles observed during acute COVID‐19 and post‐COVID symptoms and support further research into the role of serum biomarkers in post‐COVID prognostication.

## AUTHOR CONTRIBUTIONS

**Dominic L. Sykes**: Conceptualization; formal analysis; investigation; methodology; writing—original draft; writing—review and editing. **Christina M. Van der Feltz‐Cornelis**: Acquisition; methodology; supervision; writing—review and editing. **Luke Holdsworth**: Formal analysis; validation; writing—original draft; writing—review and editing. **Simon P. Hart**: Conceptualization; writing—review and editing. **Joseph O'Halloran**: Data curation; formal analysis; methodology. **Steve Holding**: Conceptualization; data curation. **Michael G. Crooks**: Conceptualization; supervision; writing—original draft; writing—review and editing.

## CONFLICT OF INTEREST STATEMENT

The authors declare no conflict of interest.

## ETHICS STATEMENT

Data collection and analysis in this study was ratified by the Hull University Teaching Hospitals NHS Trust Clinical Governance Committee, Case number: 2021.099.

## Supporting information

Supporting information.

## Data Availability

The data set for this study will be made freely available upon reasonable request.
