# Supplementary material for: Examining the relationship between inflammatory biomarkers during COVID‐19 hospitalization and subsequent long‐COVID symptoms: A longitudinal and retrospective study
Source: Immun Inflamm Dis. 2023 Oct 30;11(10):e1052. doi: 10.1002/iid3.1052 (PMC10614127; doi:10.1002/iid3.1052)
Supplement: Supplementary file 1 — Supporting information. [file IID3-11-e1052-s001.docx]

**Examining the relationship between inflammatory biomarkers during COVID-19 hospitalisation and subsequent Long-COVID symptoms: A longitudinal and retrospective study – Supplemental Materials**


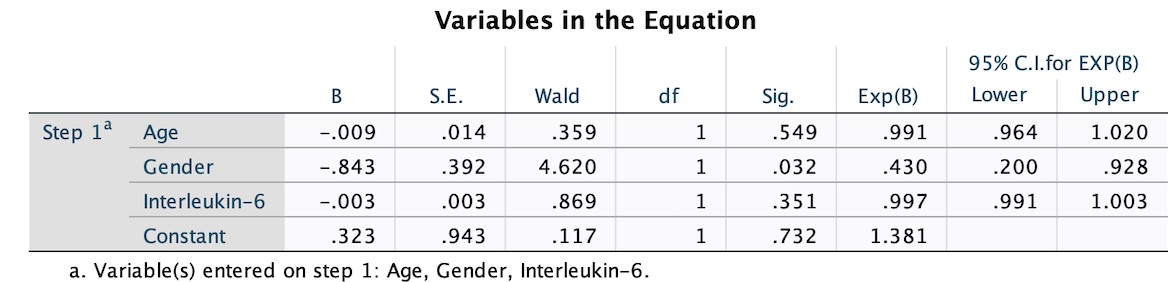


**Supplemental Table 1** *Multivariate regression analysis observing the effect of IL-6 on myalgia, adjusted for age and gender.*


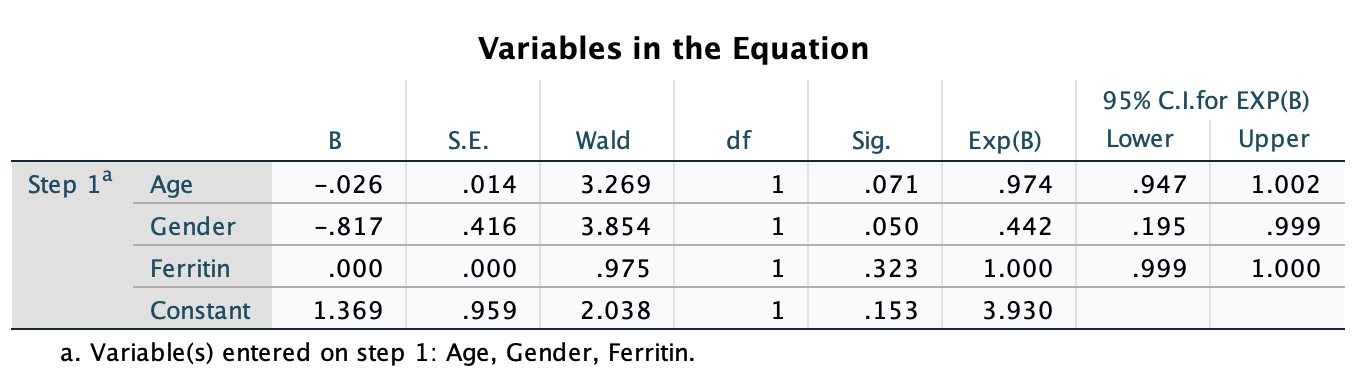


**Supplemental Table 2** *Multivariate regression analysis observing the effect of Ferritin on anxiety, adjusted for age and gender.*
